# Making a Fortune

**Published:** 1874-12

**Authors:** 


### MAKING A FORTUNE.

Mr. Joel McComber, whose writings on his improvements in the construction of lasts, on which clothing for the feet are made, have lately had a place in our columns, is full of enthusiasm concerning the interest which has thus been excited in the minds of cultivated persons all over the country. Inquiries and orders come in by every mail, and his business is extending in all directions. He finds abundant cause for satisfaction in the fact that our Journal has looked him up and given prominence to his great invention. We are as well pleased at this as he can possibly be, because we recognize the fact that he is a genuine reformer in a matter of the greatest public importance. His boots and shoes are at once the most elegant we have seen, and the only ones, so far known, which are constructed on true principles. We are glad to know that he is making a fortune through our influence. The testimony of Mr. McComber as to the value of the Journal, as a medium of communication with the best class of people, is the testimony of all. We have a large and very choice circulation. The Journal goes into the hands only of the intelligent and thoughtful, who have the brains to comprehend a great thought, the persistency to follow it up, and the financial ability to utilize it. The present number will go into 25,000 worthy families, and will be read by not less than 100,000 intelligent people.

